# PD-L1-Mediated Immunosuppression in Glioblastoma Is Associated With the Infiltration and M2-Polarization of Tumor-Associated Macrophages

**DOI:** 10.3389/fimmu.2020.588552

**Published:** 2020-11-30

**Authors:** Zhiyuan Zhu, Hongbo Zhang, Baodong Chen, Xing Liu, Shizhong Zhang, Zhitao Zong, Mengqi Gao

**Affiliations:** ^1^ Department of Functional Neurosurgery, Zhujiang Hospital, Southern Medical University, The National Key Clinical Specialty, The Engineering Technology Research Centre of Education Ministry of China, Guangdong Provincial Key Laboratory on Brain Function Repair and Regeneration, Guangzhou, China; ^2^ Division of Neurosurgery, Department of Surgery, Li Ka Shing Faculty of Medicine, The University of Hong Kong, Hong Kong, Hong Kong; ^3^ Department of Neurosurgery, Peking University Shenzhen Hospital, Shenzhen, China; ^4^ Department of Neurosurgery, Beijing Tiantan Hospital, Capital Medical University, Beijing, China; ^5^ Chinese Glioma Genome Atlas Network, Beijing, China; ^6^ Department of Neurosurgery, Jiujiang Hospital of Traditional Chinese Medicine, Jiujiang, China

**Keywords:** immune checkpoint inhibitor, PD-1/PD-L1 axis, glioblastoma tumor microenvironment, immunosuppression, tumor-associated macrophages

## Abstract

There has been no significant improvements for immune checkpoint inhibitors since its first use. Tumour-associated macrophages (TAMs) are critical mediators in the PD-1/PD-L1 axis, contributing to the immunosuppressive tumour microenvironment. This study aims to investigate the potential role of PD-L1 in regulating TAMs in glioblastoma. Gene expression data and clinical information of glioma patients were collected from TCGA (n = 614) and CGGA (n = 325) databases. Differentially expressed genes between PD-L1^high^ and PD-L1^low^ groups were identified and subjected to bioinformatical analysis. We found that PD-L1 was frequently expressed in gliomas with a grade-dependent pattern. Higher PD-L1 expression predicted shorter overall survival. Moreover, PD-L1 was positively correlated with immunosuppressive cells (macrophage, neutrophil and immature DC) and negatively correlated with cytocidal immune cells (CD8^+^ T cell and Th1). Importantly, PD-L1 high expression was significantly correlated with M2-polarization of macrophages (M2-TAMs). We conclude that PD-L1 is an unfavourable prognostic marker for patients with glioblastoma; PD-L1-mediated immunosuppression may attribute to the infiltration and M2-polarization of TAMs.

## Introduction

Glioblastoma multiforme (GBM) is the most common malignant primary brain tumor in adults. It accounts for 30% of all the brain tumors and more than 50% of gliomas (1, 2). The current standard of care for GBM patients includes surgical tumor resection followed by radiotherapy with concomitant and adjuvant temozolomide (3). Even with aggressive and comprehensive treatment, tumor recurrence is inevitable. The median overall survival of patients diagnosed with GBM is less than two years (1). Given the poor survival of GBM patients and inefficiency of the current therapy regimen, alternative treatment strategies, and novel therapeutic targets are clearly needed.

Immunotherapy is a revolutionary anti-cancer therapy in the past decade. Various immunotherapy modalities have been established, including immune checkpoint inhibitors, CAR-T, vaccine and oncolytic virus (4). The most well-known checkpoint inhibitors are antibodies of cytotoxic T lymphocyte antigen 4 (CTLA-4), programmed cell death protein 1 (PD-1), and its ligand programmed death-ligand 1 (PD-L1) (5). Upon activation, the checkpoints induce inhibitory or even apoptotic effects within immune cells (mainly effector T cells) (6, 7). Immune-checkpoint inhibitors can block the interaction between the ligands and the immune-repressive receptors, thus overcome the inhibition of immune cells and reactivate the cytocidal immune response (8). Currently, immune-checkpoint inhibitors have shown remarkable benefits in prolonging survival in many cancers such as lung cancer, breast cancer, and melanoma (9–11).

In GBM, however, therapeutic response to checkpoint inhibitors is variant and mostly ineffective. All the large phase 3 clinical studies on PD-1 inhibitors in GBM have failed to show survival benefits (12, 13). Less than 10% of patients with recurrent GBM respond to PD-1 inhibitors (14). It is recognized that GBM is highly genetically heterogeneous and unresponsive to immunotherapy approaches (15). The underlying mechanisms remain elusive and can be multifaceted, wherein the immunosuppressive tumor microenvironment (TME) represents a critical factor (13). The engagement of PD-1 and PD-L1 is an essential mechanism that contributes to the immune-suppressive TME. Multiple suppressive effects can be triggered, such as the induction of cellular apoptosis, the impairment of T lymphocyte proliferation, and the inhibition of “effector” cytokine generation (7). The majority of PD-L1 expression is contributed by tumor-infiltrating myeloid cells (TIMs, including TAM, tumor associated neutrophils and myeloid-derived suppressor cells) (16). Recent studies reported that PD-L1 inhibitor could skew TAMs towards a pro-inflammatory M1 status (17, 18), expanding its canonical T cell suppression function. Currently, the relationship between PD-L1 and TAMs in GBM remains poorly understood.

The dissatisfied efficacy of anti-PD-1 antibodies in GBM necessitates the basic research on PD1/PD-L1 axis-mediated immune resistance. This study aims to delineate the role of PD-L1 in the immunosuppressive TME, focusing on its relationship with TAMs. By using transcriptional gene expression data from CGGA (The Chinese Glioma Genome Atlas) and TCGA (The Cancer Genome Atlas) databases, we depicted the immune landscape associated with PD-L1 in GBM and proposed that PD-L1-mediated immunosuppression may correlate with the infiltration and M2-polarization of macrophages.

## Methods and Materials

### Data Acquisition

Glioma gene expression profile and patients’ clinical information were downloaded from TCGA (http://cancergenome.nih.gov) and CGGA (http://www.cgga.org.cn) databases. In the TCGA dataset, the transcriptome expression data of 150 GBM and 464 lower gliomas were collected. In the CGGA dataset, the total number of samples is 325, including 144 GBM and 181 lower gliomas. Patients’ information on age, gender, World Health Organization (WHO) grade, diagnosis with molecular characteristics, treatment, and patient prognoses were organized (**Table S1**). PD-L1 and other gene expression profiles were obtained as described previously (19, 20). Gene expression level was presented as PRKM (reads per kilobase transcriptome per million reads).

### Bioinformatic Analysis

Genes that showed consistent differential expression in both TCGA and CGGA cohorts were extracted. Overlapped highly expressed genes in the PD-L1^high^ (807 over-expressed genes) and PD-L1^low^ (559 over-expressed genes) GBMs were subjected to KEGG enrichment analysis (**Table S2**). Gene annotation and pathway enrichment analysis were performed by Database for Annotation, Visualization, and Integrated Discovery (DAVID), and Kyoto Encyclopedia of Genes and Genomes (KEGG, http://www.kegg.jp/kegg/pathway.html). Gene Set Variation Analysis (GSVA package of R http://www.bioconductor.org/) was used to explore the correlation between PD-L1 and the predefined, highly distinctive transcriptional profile of each immune cell type (21–24). The classical chemokines and surface markers of both M1- and M2- macrophages were also included (25–28). Twenty-six types of immune cells with corresponding gene signatures were utilized for analyses (**Table S3**).

### Statistical Analysis

R language (v. 3.4.3, AT&T BellLaboratories, Lucent Technologies), SPSS software (v. 22.0, IBM company), and GraphPad Prism (v. 8.0, LLC) for Windows were used for statistical analyses and figure generation. Samples from the TCGA and CGGA datasets were analysed separately. Genes differentially expressed between PD-L1 high and low groups (PD-L1^high^ and PD-L1^low^, stratified by median value) were estimated by a two-tailed Student’s t-test. The Significance Analysis of Microarrays (SAM) package of R was performed to control the FDR (False Discovery Rate). Statistically significant was considered when FDR <0.01 and norm p <0.05. The prognostic value of these differentially expressed genes was evaluated by the survival package of R. A multivariate Cox proportional hazard model was performed to evaluate the independent prognostic variables. Kaplan-Meier curves were utilized to depict the survival distributions. PD-L1-correlated genes and immune cells were explored by Pearson’s correlation coefficient (r) using R. A significant correlation was indicated by an absolute r-value greater than 0.3 and a p-value less than 0.05.

## Results

### PD-L1 Is Frequently Expressed in Gliomas and Predicts Unfavorable Overall Survival in GBM

**Figure 1:**
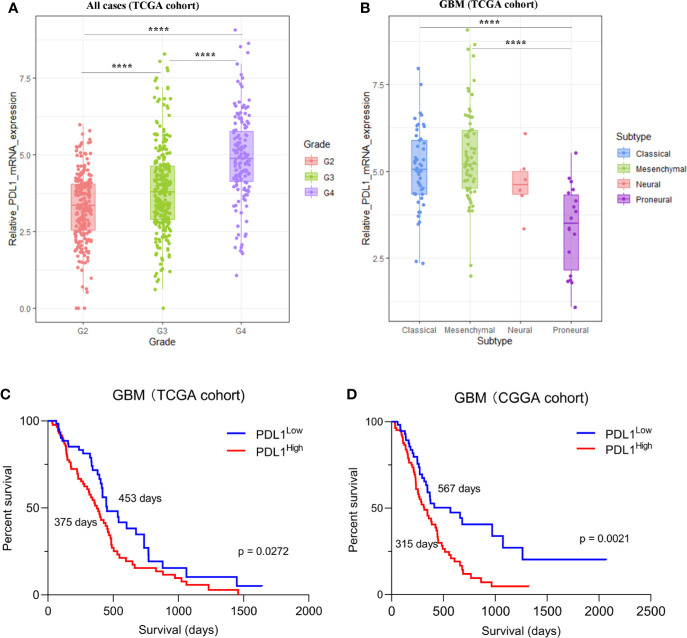
The expression of PD-L1 in gliomas in TCGA dataset. **(A)**. The expression of PD-L1 in each grade of glioma, G2 (Grade II) mean = 3.321, G3 (Grade III) mean = 3.870, G4 (Grade IV) mean = 4.915. **(B)**. Expression of PD-L1 in GBM subtypes, proneural mean = 3.365, neural mean = 4.669, classical mean = 5.078, mesenchymal mean = 5.442; one-way ANOVA followed by Tukey’s multiple comparisons test was used. **(C, D)**. Kaplan-Meier analysis showed the prognosis value of PD-L1 in GBM, median survival days of PD-L1^high^ vs. PD-L1^low^: 375 days vs 453 days (C, TCGA cohort) and 315 days vs 567 days (D, CGGA cohort); Log-rank test was used; ****p < 0.0001.

Firstly, we found that PD-L1 mRNA was frequently expressed in different grades of gliomas with a grade-dependent pattern in TCGA cohorts (**Figure 1A**). A similar trend was found in CGGA cohort, although the difference was of no statistical significance (**Figure S1A**). Of note, when compared with other GBM subtypes, the proneural subtype has particularly lower PD-L1 expression whereas the mesenchymal subtype has a relatively higher level (**Figure 1B**, **S1B**). In both cohorts, GBM patients with higher PD-L1 expression had shorter overall survival (**Figure 1C** TCGA, median survival: PD-L1^high^ vs PD-L1^low^, 375 vs 453 days, p = 0.0272; **Figure 1D** CGGA, median survival: PD-L1^high^ vs PD-L1^low^, 315 vs 567 days, p = 0.0021). Cox regression analysis further showed that PD-L1 was an independent prognostic marker in GBM (**Table S4**). The results suggested that PD-L1 was frequently expressed in gliomas and could serve as a prognostic biomarker in GBM.

### PD-L1 Is Positively Correlated With Immunosuppressive M2-Macrophage and Suppresses Effector T Cells in GBM

**Figure 2:**
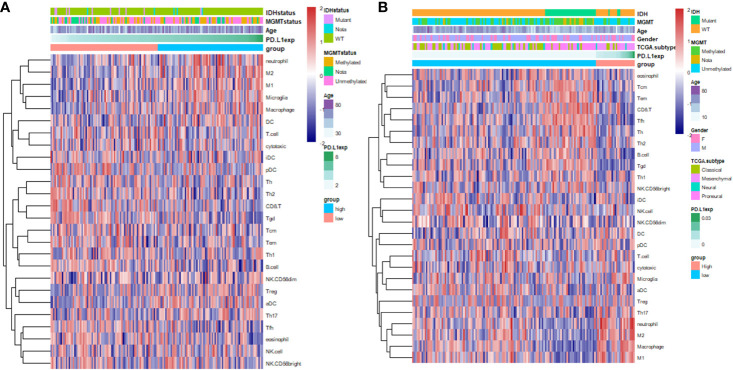
Heatmap indicates the correlation of PD-L1 with 26 immune cell subpopulations in CGGA dataset **(A)** and TCGA dataset **(B)**. Each coloured square illustrates the correlation between PD-L1 and the transcriptional profile of the corresponding immune cell type. Red colour illustrates a positive correlation (r = 1), white indicates no correlation (r = 0), and blue a negative correlation (r = −1). Immune cells in the ranking list: **(A)**, eosinophil, Tcm, Tem, CD8^+^T cell, Tfh, Th, Th2, B cell, Tgd (γδT cell), Th1, NK CD56bright cell, iDC, NK cell, NK CD56dim cell, DC, pDC, T cell, Cytotoxic T cell, Microglia, aDC, Treg, Th17, neutrophil, M2-like-Macrophage, Macrophage,M1-like-Macrophage; **(B)**, neutrophil, M2-like-Macrophage, M1-like-Macrophage, Microglia, Macrophage, DC, T cell, Cytotoxic T cell, iDC, pDC, Th, Th2, CD8^+^T cell, Tgd, Tcm, Tem, Th1, B cell, NK CD56dim cell, Treg, aDC, Th17, Tfh, eosinophil.

PD-L1 is the ligand of the well-known immune checkpoint PD-1, which mediates the suppression on effector T cell (7). To investigate the immune cells that may correlate with PD-L1 in GBM, we identified PD-L1-associated immune components in GBM microenvironment by utilizing GSVA analysis (**Figure 2** and **Table S5**). The results showed that PD-L1 expression was positively correlated with the infiltration of macrophages (CGGA r = 0.3024, p = 0.0002; TCGA r = 0.30818, p = 0.0001), M1-macrophage (CGGA r = 0.25567, p = 0.002; TCGA r = 0.36842, p < 0.0001), M2-macrophage (CGGA r = 0.30057, p = 0.0003; TCGA r = 0.36069, p < 0.0001), neutrophil (CGGA r = 0.33619, p < 0.0001; TCGA r = 0.26032, p = 0.0013), and Treg (CGGA, r = 0.02204, p > 0.05; TCGA, r = 0.21973, p = 0.0069). In contrast, PD-L1 was negatively correlated with the infiltration of CD8^+^ T cell (CGGA, r = -0.19901, p = 0.0168; TCGA, r = -0.35739, p < 0.0001), Tfh (CGGA, r = -0.3092, p = 0.0002; TCGA, r = -0.25058, p = 0.0020), Tgd (CGGA, r = -0.30892, p = 0.0002; TCGA, r = -0.36625, p < 0.0001), and B cell (CGGA r = -0.32434, p < 0.0001; TCGA r = –0.26846, p = 0.0009). These results indicated that PD-L1 was positively correlated with the infiltration of macrophages in GBM and negatively correlated with effector T immune cells.

### PD-L1 Correlates With M2-Macrophages-Related Chemokines

**Figure 3:**
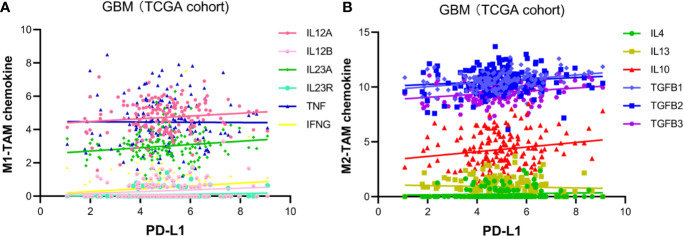
Pearson’s correlation of PD-L1 and TAM-related chemokines in TCGA glioblastoma dataset. **(A)** Chemokine of M1-TAM: IL12A (r = 0.117796, p > 0.05), IL12B (r = 0.140694, p > 0.05), IL23A (r = 0.149971, p > 0.05), IL23R (r = 0.107516, p > 0.05), TNF (r = -0.00741, p > 0.05), IFNG (r = 0.129588, p < 0.0001). **(B)** Chemokine of M2-TAM: TGF-β1 (r = 0.34433, p < 0.0001), TGF-β2 (r = 0.12637, p = 0.1148), TGF-β3 (r = 0.22328, p < 0.0049), IL-10 (r = 0.18208, p = 0.0225), IL-4 (r = 0.0748, p > 0.05), IL-13 (r = -0.05977, p > 0.05).

The M2-polarization of TAM is recognized as an immune-suppressive phenotype (29). It is striking to notice the correlation between PD-L1 and M2-macrophages. To verified the above findings, we investigated the correlation between PD-L1 and canonical chemokines of both M1-macrophages (IL-12, IL-23, TNF, IFNG) and M2 macrophages (IL-10, TGF-β, IL-4, IL-13). In TCGA cohort (**Figure 3**), PD-L1 was positively correlated with M2-macrophage chemokines, such as TGF-β1 (r = 0.34433, p < 0.0001), TGF-β3 (r = 0.22328, p = 0.0049), and IL-10 (r = 0.18208, p = 0.0225). However, no significant correlation was found between PD-L1 and M1-macrophage chemokines (|r| < 0.1, p > 0.05). In CGGA cohort, PD-L1 also showed positive correlation with M2 chemokines, although the differences were not statistically significant (**Figure S2B**). These findings supported that PD-L1 was intimately correlated with M2-macrophages in the microenvironment of GBM.

### PD-L1 Is Associated With Signalling Pathways That Modulate Macrophage Polarization

**Figure 4:**
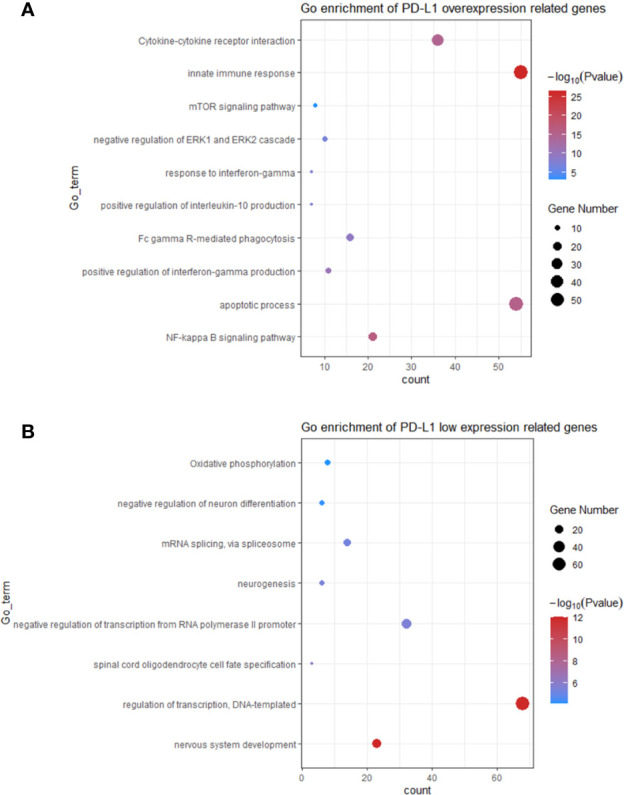
The top bioinformatics hits of biological pathways derived from genes enriched in GBM patients with PD-L1^high^ **(A)** and PD-L1^low^ **(B)**. Plot sizes show gene counts enriched in the enrichment of pathway. Colour depth indicates the p value from low (red) to high level (blue). The p values of all presented hits are less than 0.05.

In order to investigate the function of PD-L1 in modulating TAM M2 polarization, genes highly expressed in PD-L1^high^ and PD-L1^low^ GBMs were used to identify potential biological pathways (**Table S2**). As shown in **Figure 4**, the pathways enriched in PD-L1^high^ GBM include (1) NF-kappa B signalling pathway; (2) apoptotic process; (3) positive regulation of interferon-gamma production; (4) Fc gamma R-mediated phagocytosis; (5) positive regulation of interleukin-10 production; (6) response to interferon-gamma; (7) negative regulation of ERK1 and ERK2 cascade; (8) mTOR signalling pathway; (9) innate immune response; (10) Cytokine-cytokine receptor interaction. Enriched pathways in PD-L1^low^ GBM contain (1) nervous system development; (2) regulation of transcription, DNA-templated; (3) spinal cord oligodendrocyte cell fate specification; (4) negative regulation of transcription from RNA polymerase II promoter; (5) neurogenesis; (6) mRNA splicing, *via* spliceosome; (7) negative regulation of neuron differentiation; (8) Oxidative phosphorylation. In sum, critical pathways involved in macrophage polarization were enriched in PD-L1^high^ GBMs, while biological pathways enriched in PD-L1^low^ GBMs were less relevant with macrophages functions.

## Discussion

The continuous failure of clinical trials on PD-1 antibodies in GBM necessitates basic researches on the mechanism of immunotherapies resistance. This study depicts the immune features associated with PD-L1 in the TME of GBM. Firstly, the PD-L1 mRNA expression shows a grade-dependent pattern in gliomas. Higher PD-L1 expression predicted a poorer outcome in patients with GBM. Moreover, PD-L1 expression is associated with the infiltration of immune-suppressive macrophages and neutrophils. We further found that PD-L1 high expression was positively correlated with the M2-polarization of TAMs, evidenced by the increased M2-related gene signatures and canonical chemokines. Signalling pathways that correlated with macrophage polarization were enriched in PD-L1^high^ GBMs, indicating a critical role of PD-L1 in modulating macrophage activation. The present study provides preliminary evidence on the intimate correlation between PD-L1 and M2-TAMs, supporting the notion that PD-L1 inhibitors could enhance the efficacy of prevalent PD-1 antibodies for GBM therapy.

It is important to determine the expression pattern of PD-L1 in GBM. The protein level of PD-L1 has been considered as a critical predictive marker for therapeutic response to PD-1/PD-L1 antibody in multiple types of cancer (30). However, the positive rate and expression level of PD-L1 in GBM can be influenced by many factors, such as the selected anti-PD-L1 antibody; the positive criteria; and the intrinsic tumoral heterogeneity (31). For instance, the percentage of GBM patients with detectable PD-L1 protein expression level varies from 61 to 88% according to different reports (32, 33), while the median percentage of PD-L1-expression cells in GBM is only 2.77% (32). Thus, a more comprehensive landscape of PD-L1 expression in glioma is needed. In this study, we found that PD-L1 mRNA was frequently expressed in all grades of gliomas and exhibited a grade-dependent manner. This finding is in line with previous studies that PD-L1 is positively correlated with glioma grades (34). We also noticed that the proneural GBM subtype had lower PD-L1 expression among all the GBM subgroups whereas the mesenchymal subtype had a relatively higher level. These findings are in agreement with other reports that the proneural subtype has a better outcome and the immunosuppressive genes are predominant in mesenchymal subtype (35, 36).

Whether PD-L1 represents a stable prognosis predictor in glioma is still under debate. Over half of the published reports proposed the negative correlation of PD-L1 expression and survival time of glioma patients, while other studies showed no significant correlation between PD-L1 and patient survival (31, 33, 37). This study shows that higher PD-L1 mRNA expression is correlated with shorter overall survival. The Cox regression analysis further indicates that PD-L1 is an independent unfavourable prognostic marker in GBMs.

Intra-tumor heterogeneity and unresponsive to immunotherapy represent the major obstacles for immune-checkpoint antibodies in GBM. The WHO 2016 glioma diagnosis scheme based on molecular characteristics represents a big step towards precise diagnosis and tailored therapy for patients with diffused glioma (38). GBMs are well-known insensitive “cold” tumors with relatively low tumor mutation burden and quiescent immune reactivity (13, 39). The highly immune-suppressive TME with a paucity of infiltrating CTLs has been considered a pivotal mediator of the insensitivity (40), wherein TAMs play an indispensable role (16). Classically, TAMs can polarize to M1 macrophages (the classical activation) which exhibit pro-inflammatory and cancer-inhibiting effects. Alternatively, stimuli such as IL-4, IL-14, IL-10 can induce macrophages towards an anti-inflammatory and cancer-promoting M2 phenotype (41, 42). In GBM, TAMs were the predominant infiltrating immune cells and usually polarized to an immunosuppressive M2-like phenotype (43, 44). This study shows that PD-L1 may be an important regulator of macrophages in GBM, supported by the positive correlation between them. Moreover, PD-L1 is positively correlated with the canonical markers of M2 macrophages, while has insignificant correlation with M1 markers. Thus, PD-L1 may participate in the induction and maintenance of macrophage M2-polarization.

The tumor infiltrating neutrophils could also inhibit T cells activity *via* the PD-1/PD-L1 pathway in hepatocellular carcinoma (45) and was associated with pro-inflammatory cytokine in lung cancer (46). The positive correlation between PD-L1 and neutrophil in the study implies that neutrophil-mediated immunosuppression may also occur in GBM. Regulatory T cells (Treg) are emerging as a mediator of immunosuppression in glioblastoma by inhibiting the generation of IL-12 and IFN-γ and suppressing tumor infiltrating T cells (47). PD-L1-mediated immunosuppression may also involve Treg.

In contrast, PD-L1 shows negative correlations with CD8^+^ cytotoxic T cells, T follicular helper cells (Tfh), gamma delta (γδ) T cells (Tgd), and B cells. CD8^+^ T cells represent the major tumoricidal T lymphocyte. The immunosuppressive TME is feathered by the exhaustion, anergy, and apoptosis of CD8^+^ T cells (48). Tfh belongs to CD4^+^ T cells which play a critical immune protective role *via* facilitating B cell generating antibody (49). Tgd produces various cytokines and chemokines (IFN-γ, TNF-α, IL-17) and can lysate infected or malignant cells (50). Thus, the immunosuppression mediated by PD-L1 may involve in a broader range of anti-tumor lymphocytes.

Genes that highly expressed in PD-L1^high^ GBMs enriched in multiple critical polarizing-related signalling pathways. For instance, The classical activation of macrophages (M1) is induced by LPS and interferon-gamma (IFN-γ) (51). NF-kappa B is a critical transcription factor that transduces activation signals from LPS/TLR4 (52). Meanwhile, Shen J *et al.* also showed that NF-kappa B mediated IL-17 induced M2 macrophage polarization (53). IL-10 represents one of the canonical M2 stimuli. Upon ligating with its receptor IL-10R, IL-10 activates the transcription factor STAT3, which suppresses the production of pro-inflammatory cytokine (54, 55). The key role of mTORC1 in macrophage polarization has been reported, constitutive mTORC1 activation impaired the M2 polarization and increase pro-inflammatory response (56); depletion of mTORC1 decreased inflammation and pathogenic immune response during infection (57).

Translationally, targeting PD-L1 may represent a promising approach to re-educate TAM towards the anti-tumor M1 phenotype. Inhibition of PD-L1 would, therefore, abrogate the immune-suppression induced by M2 macrophage. It has been reported that PD-L1 involved in constitute negative signals pathways and led to immune-suppressive phenotypes in macrophages. Inhibition of PD-L1 promoted the proliferation, survival, and activation of macrophages (18). A similar remodeling of intratumoral macrophages was observed in a murine sarcoma model treated with anti-PD1/anti-CTLA-4 antibodies (17). Our study and others imply that targeting the PD-1/PD-L1 axis may yield additional anti-cancer effects mediated by TAM polarization. This notion can be exploited in the treatment of GBM since the combinations of PD-L1 and PD-1 antibodies have shown potent anti-GBM efficacy in pre-clinical studies (58–60).

Experimental studies are needed to validate the findings of this study. *Ex vivo* profiling of the cell components in GBM tissues would provide valuable information of TME. In addition, the correlation analysis can only provide preliminary evidence of the relationship rather than determine the causal relationship between PD-L1 and M2 macrophages. One of the major challenges of exploiting PD-L1 for prognosis prediction in GBM is to determine the positive criteria and cut-off value of PD-L1 expression, which necessitates large-scale studies. Lastly, it remains elusive whether M2-TAM polarization causally induces PD-L1 expression or vice versa, further studies are needed to delineate the relationship between PD-L1 and macrophage polarization.

This study provides preliminary evidence that PD-L1 is intimately correlated the infiltration and M2-polarization of macrophages. This notion represents an underappreciated immunosuppressive mechanism in the context of GBM. One brave but reasonable speculation is that PD-L1 inhibitors may enhance the efficacy of the prevalent PD-1 antibodies in GBM. It would be worthy to evaluate the efficacy of combining PD-L1 and PD-1 antibodies in future clinical trials.

## Data Availability Statement

The original contributions presented in the study are included in the article/**Supplementary Material**. Further inquiries can be directed to the corresponding authors.

## Author Contributions

ZYZ and MG designed the study and wrote the manuscript. HZ designed the study, interpreted the results, and revised the manuscript. XL and MG performed the analysis and prepared the figures. BC, SZ, and ZTZ designed and supervised the study. All authors contributed to the article and approved the submitted version.

## Funding

This work was supported by the funding: Basic research of Shenzhen Science and technology plan project (No. JCYJ2017306091310788).

## Conflict of Interest

The authors declare that the research was conducted in the absence of any commercial or financial relationships that could be construed as a potential conflict of interest.
