# Supplementary material for: PD-L1-Mediated Immunosuppression in Glioblastoma Is Associated With the Infiltration and M2-Polarization of Tumor-Associated Macrophages
Source: Front Immunol. 2020 Nov 30;11:588552. doi: 10.3389/fimmu.2020.588552 (PMC7734279; doi:10.3389/fimmu.2020.588552)
Supplement: Supplementary file 1 [file Table_1.docx]

Table S1. Baseline demographic and clinical characteristics of the patients in this study

|  | n (%) |
| --- | --- |
| ***TCGA cohort*** |  |
| *Gender* |  |
| Male | 353 (57.5) |
| Female | 254 (41.4) |
| *Age (years at diagnosis)* |  |
| > 45 | 317 (51.6) |
| < = 45 | 290 (47.2) |
| *Grade* |  |
| GBM | 150 (24.4) |
| LGG | 464 (75,6) |
| *IDH mutation* |  |
| IDH mutant | 386 (62.9) |
| IDH wild-type | 221 (36.0) |
| *MGMT promotor methylation* |  |
| MGMT unmeth | 146 (23.8) |
| MGMT meth | 436 (71.0) |
| *1p-19q codeletion* |  |
| codel | 155 (25.2) |
| Non-codel | 453 (73.8) |
| *Total* | 614 (100) |
| ***CGGA cohort*** |  |
| *Gender* | GBM |
| Male | 203 (62.5) |
| Female | 122 (37.5) |
| *Age (years at diagnosis)* |  |
| > 45 | 125 (38.5) |
| < = 45 | 200 (61.5) |
| *Grade* |  |
| GBM | 144 (44.3) |
| LGG | 181 (55.7) |
| *IDH mutation* |  |
| IDH mutant | 167 (51.4) |
| IDH wild-type | 158 (48.6) |
| *MGMT promotor methylation* |  |
| MGMT unmeth | 117 (36.0) |
| MGMT meth | 139 (42.8) |
| *1p-19q codeletion* |  |
| codel | 69 (21.2) |
| Non-codel | 256 (78.8) |
| *Total* | 325 (100) |

n. number of patients
