# Supplementary material for: PD-L1-Mediated Immunosuppression in Glioblastoma Is Associated With the Infiltration and M2-Polarization of Tumor-Associated Macrophages
Source: Front Immunol. 2020 Nov 30;11:588552. doi: 10.3389/fimmu.2020.588552 (PMC7734279; doi:10.3389/fimmu.2020.588552)
Supplement: Supplementary file 4 [file Table_4.docx]

Table S4. Correlation of PD-L1 and common clinicopathological factors with patients’ overall survival in the CGGA and TCGA datasets

| **Variables** | **Univariate analysis** | | | **Multivariate analysis** | | |
| --- | --- | --- | --- | --- | --- | --- |
|  | **HR** | **95%CI** | **P value** | **HR** | **95%CI** | **P value** |
| ***TCGA*** |  |  |  |  |  |  |
| ***All patients*** |  |  |  |  |  |  |
| **PDL1** | **1.786** | **1.602 - 1.992** | **0.000*** | **1.397** | **1.218 - 1.602** | **0.000*** |
| Age | 1.076 | 1.064 - 1.089 | 0.000* | 1.059 | 1.042 - 1.075 | 0.000* |
| Gender | 0.994 | 0.738 - 1.338 | 0.967 | 1.172 | 0.825 - 1.663 | 0.376 |
| Grade | 4.855 | 3.784 - 6.229 | 0.000* | 1.656 | 1.206 - 2.275 | 0.002* |
| IDH mutation | 0.096 | 0.068 - 0.137 | 0.000* | 0.473 | 0.254 - 0.880 | 0.018* |
| 1p19q co-deletion | 0.222 | 0.131 - 0.378 | 0.000* | 0.639 | 0.341 - 1.198 | 0.163 |
| MGMT methylation | 0.325 | 0.233 - 0.451 | 0.000* | 0.755 | 0.504 - 1.132 | 0.174 |
| ***GBM*** |  |  |  |  |  |  |
| **PDL1** | **1.148** | **0.989 - 1.332** | **0.069** | **1.291** | **1.051 - 1.586** | **0.015*** |
| Age | 1.038 | 1.018 - 1.058 | 0.000* | 1.054 | 1.025 - 1.083 | 0.000* |
| Gender | 1.200 | 0.800 - 1.800 | 0.378 | 1.319 | 0.737 - 2.362 | 0.351 |
| IDH mutation | 0.276 | 0.100 - 0.759 | 0.013* | 0.748 | 0.161 - 3.488 | 0.712 |
| 1p19q co-deletion | - | - | - | - | - | - |
| MGMT methylation | 0.690 | 0.424 - 1.123 | 0.136 | 0.673 | 0.383 - 1.183 | 0.169 |
| ***CGGA*** |  |  |  |  |  |  |
| ***All patients*** |  |  |  |  |  |  |
| **PDL1** | **9e+23** | **9e+10 - 8e+36** | **0.000*** | **3.8e+21** | **5e+6 - 2e+34** | **0.000*** |
| Age | 1.038 | 1.023 - 1.054 | 0.000* | 1.007 | 0.991 - 1.024 | 0.390 |
| Gender | 0.847 | 0.600 - 1.195 | 0.345 | 0.757 | 0.518 - 1.105 | 0.149 |
| Grade | 3.477 | 2.716 - 4.452 | 0.000* | 2.304 | 1.705 - 3.113 | 0.000* |
| IDH mutation | 0.228 | 0.158 - 0.329 | 0.000* | 0.733 | 0.433 - 1.242 | 0.249 |
| 1p19q co-deletion | 0.135 | 0.068 - 0.267 | 0.000* | 0.290 | 0.126 - 0.664 | 0.003* |
| MGMT methylation | 0.526 | 0.371 - 0.745 | 0.000* | 0.742 | 0.503 - 1.069 | 0.134 |
| ***GBM*** |  |  |  |  |  |  |
| **PDL1** | **2e+24** | **9e+9 - 4e+38** | **0.000*** | **7.6e+21** | **8e+6 - 7e+36** | **0.004*** |
| Age | 1.005 | 0.988 - 1.022 | 0.569 | 1.004 | 0.984 - 1.024 | 0.712 |
| Gender | 0.815 | 0.528 - 1.257 | 0.355 | 0.834 | 0.525 - 1.325 | 0.442- |
| IDH mutation | 0.638 | 0.380 - 1.072 | 0.089 | 0.836 | 0.414 - 1.686 | 0.617 |
| 1p19q co-deletion | 0.563 | 0.138 - 2.290 | 0.422 | 0.889 | 0.215 - 3.680 | 0.871 |
| MGMT methylation | 0.564 | 0.364 - 0.872 | 0.010 | 0.677 | 0.410 - 1.115 | 0.125 |

HR: hazard ration, CI: confidence interval. *p<0.05.
