# Supplementary material for: PD-L1-Mediated Immunosuppression in Glioblastoma Is Associated With the Infiltration and M2-Polarization of Tumor-Associated Macrophages
Source: Front Immunol. 2020 Nov 30;11:588552. doi: 10.3389/fimmu.2020.588552 (PMC7734279; doi:10.3389/fimmu.2020.588552)
Supplement: Supplementary file 6 [file DataSheet_1.pdf]

Figure S1.

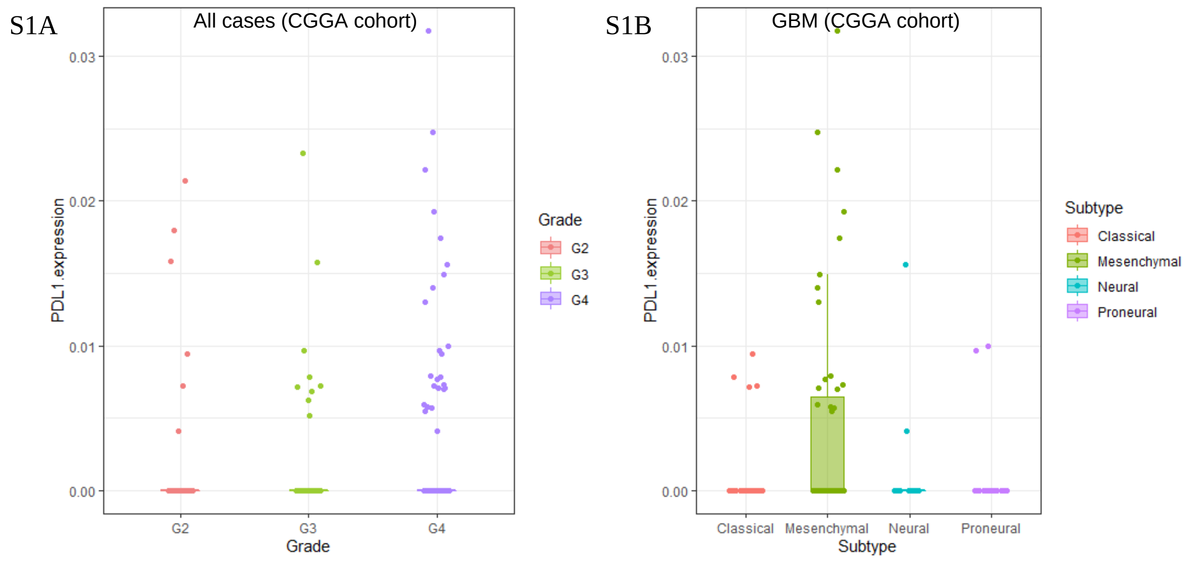

Figure S2.

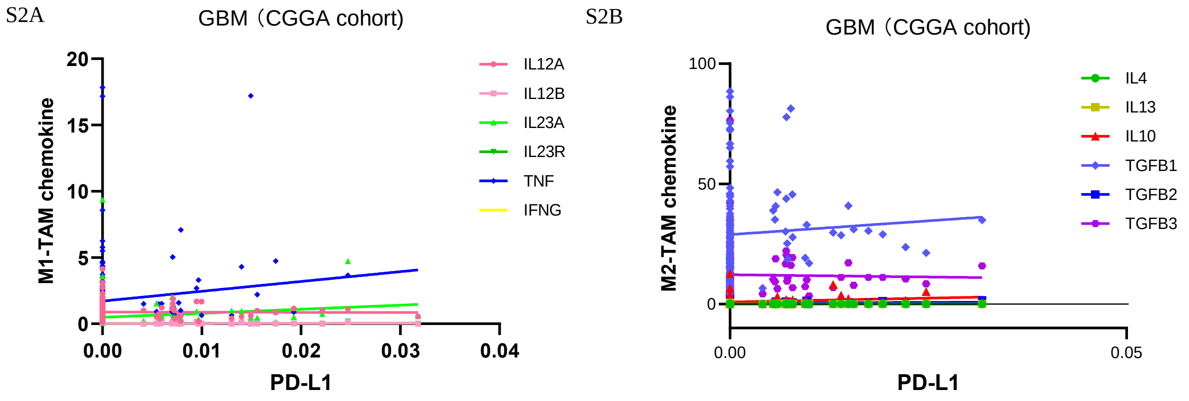

Fig. S1 Expression of PD-L1 in gliomas in CGGA dataset. A. Expression of PD-L1 in each grade of glioma; B. Expression of PD-L1 in GBM subtypes.

Fig S2 Correlation of PD-L1 and TAM-related chemokines in CGGA glioblastoma dataset. A. Chemokine of M1-TAM: IL12A ($r = -0.0099$, $p > 0.05$), IL12B ($r = 0.212959$, $p < 0.05$) , IL23A ($r = 0.174932$, $p < 0.05$), IL23R ($r = 0.042128$, $p > 0.05$), TNF ($r = 0.140619$, $p > 0.05$), IFNG ($r = -0.02935$, $p > 0.05$). B. Chemokine of M2-TAM: TGF-β1 ($r = 0.07003$, $p > 0.05$), TGF-β2 ($r = 0.15186$, $p > 0.05$), TGF-β3 ($r = -0.02224$, $p > 0.05$), IL-10 ($r = 0.22407$, $p = 0.0069$), IL-4 ($r = -0.0394$, $p > 0.05$), IL-13 ($r = -0.05202$, $p > 0.05$).
